# New Syndromes Identified by Neuroimaging during Cerebral Malaria

**DOI:** 10.4269/ajtmh.17-0926

**Published:** 2018-01-08

**Authors:** Angelika Hoffmann, Samuel C. Wassmer

**Affiliations:** 1 Department of Neuroradiology, Heidelberg University Hospital, Heidelberg, Germany;; 2 Department of Infection & Immunity, London School of Hygiene & Tropical Medicine, London, United Kingdom

Cerebral malaria (CM) is an acute nontraumatic encephalopathy, and the most severe neurological complication of *Plasmodium falciparum* infection. Mortality is high, long-term neurocognitive deficits are frequent in survivors, and the pathogenetic mechanisms leading to CM are still being debated. The application of advanced neuroimaging techniques to patients with CM has revolutionized our understanding of the disease in recent years^1^ and has allowed for the first time, the identification of brain swelling as a common occurrence in CM. In severe cases, the increase in cerebral volume is such that it results in brain stem herniation leading to death by respiratory arrest in pediatric patients.^2^ These observations have incited several investigations into the mechanisms of this pathology with the goal of identifying potential adjunct therapeutic targets.

In this issue of the *American Journal of Tropical Medicine and Hygiene*, Yamamoto et al.^3^ describe the case of a young adult who presented in Japan after returning from Kenya with falciparum malaria and subsequently developed CM. The patient underwent brain magnetic resonance time-of-flight angiography, a magnetic resonance imaging (MRI) technique to visualize flow within vessels without the need to administer contrast, and the generated images of arteries showed reversible segmental constriction. This transient vasospasm results in a diffuse “string of beads” appearance, which was observed in multiple cerebral arteries and resolved after antimalarial treatment. The findings are congruent with reversible cerebral vasoconstriction syndrome (RCVS), which the authors describe for the first time in CM.

RCVS is a clinical and radiological syndrome characterized by acute onset, recurrent headache, and reversible narrowing of multiple cerebral arteries.^4^ The neurological complications of RCVS are numerous and frequently include posterior reversible encephalopathy syndrome (PRES), another clinical–radiologic syndrome characterized by reversible, posterior-predominant vasogenic edema. This edema usually reverses completely in a few days, but cerebral infarction, cytotoxic edema, or hemorrhage can occur. Remarkably, using a 1.5 T MRI, our group recently identified a high frequency of PRES associated with increased brain volume in a case series of Indian pediatric and adult CM patients.^5^ Similar findings are reported by Potchen et al.^6^ in a Zambian pediatric case series also featured in this issue suggesting that PRES is a common occurrence in CM, irrespective of age or associated complications.

These collective findings raise several questions. First, could CM entail a combination of previously described clinical–radiologic syndromes? It is plausible that RCVS and PRES occur either independently or sequentially in CM. PRES was not reported in the case described previously, perhaps, as the authors suggested because of the late imaging of the patient.^3^ Indeed, PRES reversed rapidly in both Indian^5^ and Zambian^6^ patients, and usually resolves faster than vasoconstriction. Ongoing large-scale studies combining serial MRI and magnetic resonance angiography will soon provide valuable answers. It is likely that there is a wide spectrum of radiological presentations in CM, owing to the admission of patients at different stages of the disease. Second, could RCVS and PRES occur during fatal CM? Both syndromes are reversible by definition, and to date they have only been described in patients surviving CM. Although RCVS typically resolves spontaneously, a progressive vasoconstriction leading to a fatal outcome can develop in about 2% of cases. PRES has reported mortality rates up to 5.7%, but also usually resolves after the precipitating cause is eliminated or treated. In CM patients, the etiology of RCVS or PRES would be severe *P. falciparum* infection, and it is conceivable that these syndromes develop in fatal cases too advanced for effective antimalarial treatment. A rigorous evaluation of the association of RCVS and PRES with mortality in the context of CM is warranted. Third, because neither syndrome is directly treatable, how might their identification be relevant for clinicians who need to provide aggressive care for patients with potentially life-threatening CM? The novel documentation of RCVS and PRES during CM contributes to our understanding of the pathophysiology of the disease which, in turn, may inform new adjunct therapies that may increase patient survival. Indeed, the two conditions share many clinicoradiographic features indicating overlapping or similar pathogenetic mechanisms. Recent studies indicate that endothelial dysfunction is a common pathophysiologic factor associated with RCVS and PRES.^7,8^

The picture suggested by recent imaging findings is consistent with mounting evidence for a pivotal role of endothelial activation and dysfunction in CM. The inflammatory activation of endothelial cells after infection by *P. falciparum* is well described, and various parasite-derived factors released at sites of sequestration have been suggested to affect endothelial and blood-brain barrier (BBB) integrity. Perhaps most strikingly, recent years have seen a flurry of research activities focused on the effect of *P. falciparum*–parasitized erythrocytes binding to endothelial protein C receptor (EPCR), a newly described cytoadherence ligand in CM. The association between EPCR-binding parasite predominance and the occurrence of severe or fatal brain swelling was recently demonstrated in pediatric patients from Malawi.^9^ In addition, the authors showed that EPCR binding by parasite proteins led to a disruption of endothelial monolayer permeability in vitro. Cerebral sequestration of EPCR-specific parasites may, therefore, not only cause microvessel obstruction, but also alter BBB integrity, contributing thereby to brain swelling and, potentially, RCVS and/or PRES.

But several pieces of the puzzle are still missing. Indeed, the link between EPCR-binding parasites and endothelial dysfunction leading to vasogenic edema in PRES,^5^ or to subtle impairment of the BBB in RCVS and PRES,^7^ remains to be established in CM patients. This will prove challenging, as the diagnosis of these recently defined syndromes relies on the use of expensive high-field MRI techniques not systematically available in malaria-endemic countries. In addition, vasospasm during RCVS occurs in arteries, which are not preferred sites for sequestration of parasitized erythrocytes. The binding of EPCR-specific parasites to endothelial cells alone is therefore unlikely to account for the development of this syndrome. Another hypothesis is that free heme released by the rupture of adherent schizonts causes a transient depletion of nitric oxide and an increase in endothelin-1 expression, a powerful endogenous vasoconstrictor.^10^ The segmental narrowing in cerebral arteries could also be an upstream compensatory mechanism in response to the impaired venous drainage caused by abundant sequestration.

In conclusion, the radiographic findings presented in this issue^3,6^ show further evidence that pathological mechanisms in CM involve cerebral vascular dysregulation and highlight priorities for future neuroimaging studies of CM patients.

## References

[1] Mohanty S Taylor TE Kampondeni S Potchen MJ Panda P Majhi M Mishra SK Wassmer SC, 2014 Magnetic resonance imaging during life: the key to unlock cerebral malaria pathogenesis? Malar J 13: 276. 25038815 10.1186/1475-2875-13-276 PMC4114090

[2] Seydel KB 2015 Brain swelling and death in children with cerebral malaria. N Engl J Med 372: 1126–1137. 25785970 10.1056/NEJMoa1400116 PMC4450675

[3] Yamamoto K Kato Y Shinohara K Kutsuna S Takeshita N Hayakawa K Iwagami M Kano S Watanabe S Ohmagari N, 2018 Reversible cerebral vasoconstriction syndrome in cerebral malaria: a case report. Am J Trop Med Hyg 98: 505–507. 10.4269/ajtmh.17-0665 PMC5929209 29260652

[4] Ducros A, 2012 Reversible cerebral vasoconstriction syndrome. Lancet Neurol 11: 906–917. 22995694 10.1016/S1474-4422(12)70135-7

[5] Mohanty S 2017 Magnetic resonance imaging of cerebral malaria patients reveals distinct pathogenetic processes in different parts of the brain. mSphere 2: pii: e00193–17. 28596990 10.1128/mSphere.00193-17 PMC5463026

[6] Potchen MJ 2018 1.5 Tesla magnetic resonance imaging to investigate potential etiologies of brain swelling in pediatric cerebral malaria. Am J Trop Med Hyg 98: 497–504. 10.4269/ajtmh.17-0309 PMC5929182 29313473

[7] Lee MJ Cha J Choi HA Woo SY Kim S Wang SJ Chung CS, 2017 Blood-brain barrier breakdown in reversible cerebral vasoconstriction syndrome: implications for pathophysiology and diagnosis. Ann Neurol 81: 454–466. 28195428 10.1002/ana.24891

[8] Marra A Vargas M Striano P Del Guercio L Buonanno P Servillo G, 2014 Posterior reversible encephalopathy syndrome: the endothelial hypotheses. Med Hypotheses 82: 619–622. 24613735 10.1016/j.mehy.2014.02.022

[9] Kessler A 2017 Linking EPCR-binding PfEMP1 to brain swelling in pediatric cerebral malaria. Cell Host Microbe 22: 601–614.e5. 29107642 10.1016/j.chom.2017.09.009 PMC5783720

[10] Eisenhut M, 2015 The evidence for a role of vasospasm in the pathogenesis of cerebral malaria. Malar J 14: 405. 26463364 10.1186/s12936-015-0928-4 PMC4603731

